# Update on GPCR-based targets for the development of novel antidepressants

**DOI:** 10.1038/s41380-021-01040-1

**Published:** 2021-02-15

**Authors:** Ioannis Mantas, Marcus Saarinen, Zhi-Qing David Xu, Per Svenningsson

**Affiliations:** 1 Department of Clinical Neuroscience, Karolinska Institute, Stockholm, Sweden; 2 Department of Neurobiology, Beijing Key Laboratory of Neural Regeneration and Repair, Beijing Institute for Brain Disorders, Capital Medical University, Beijing, China

**Keywords:** Drug discovery, Depression

## Abstract

Traditional antidepressants largely interfere with monoaminergic transport or degradation systems, taking several weeks to have their therapeutic actions. Moreover, a large proportion of depressed patients are resistant to these therapies. Several atypical antidepressants have been developed which interact with G protein coupled receptors (GPCRs) instead, as direct targeting of receptors may achieve more efficacious and faster antidepressant actions. The focus of this review is to provide an update on how distinct GPCRs mediate antidepressant actions and discuss recent insights into how GPCRs regulate the pathophysiology of Major Depressive Disorder (MDD). We also discuss the therapeutic potential of novel GPCR targets, which are appealing due to their ligand selectivity, expression pattern, or pharmacological profiles. Finally, we highlight recent advances in understanding GPCR pharmacology and structure, and how they may provide new avenues for drug development.

Major depressive disorder (MDD) or Major Affective Disorder is a severe psychiatric disorder affecting over 200 million people worldwide [1] The life-time depression risk is 15–18% and is higher in women than men [1]. MDD is often life-threatening due to the illness itself with a high suicide rate and as consequence of co-morbidities, such as drug abuse disorders and cardiovascular disease. MDD is commonly defined by the two major diagnostic classification systems: the Diagnostic and Statistical Manual of Mental Disorders or International Classification of Diseases. It is based on the presence of a certain number of signs and symptoms, including feelings of guilt, hopelessness, dysphoria, cognitive dysfunction, persistent sleep, and appetite abnormalities. However, categorical symptom-based disorder diagnoses often suffer from problems with heterogeneity because of the varied neurobiological mechanisms whereby people can qualify for a diagnosis. Moreover, patients who meet criteria for one mental disorder often tend to meet criteria for other mental disorders. Such co-morbidities can complicate the choice of therapy. There are indeed several alterations in brain circuitries, neuronal networks, and molecular pathways associated with MDD resulting in neurotransmitter and neuropeptide alterations, maladaptive neuroplasticity, hypothalamus–pituitary–adrenal (HPA) axis dysfunction, abnormal immune system responses, and circadian arrhythmicity. There are currently many different approaches to treat MDD. In this review we discuss the roles of G protein coupled receptors (GPCRs) to mediate actions of existing antidepressant therapies. Based on recent insights into the biology of GPCRs, we then discuss new avenues for optimized usage of GPCRs currently targeted by antidepressants. Finally, the review discusses the therapeutic potential of novel GPCR targets which are localized in brain circuitries implicated in MDD pathophysiology.

## Brain regions and circuitries involved in MDD

**Fig. 1:**
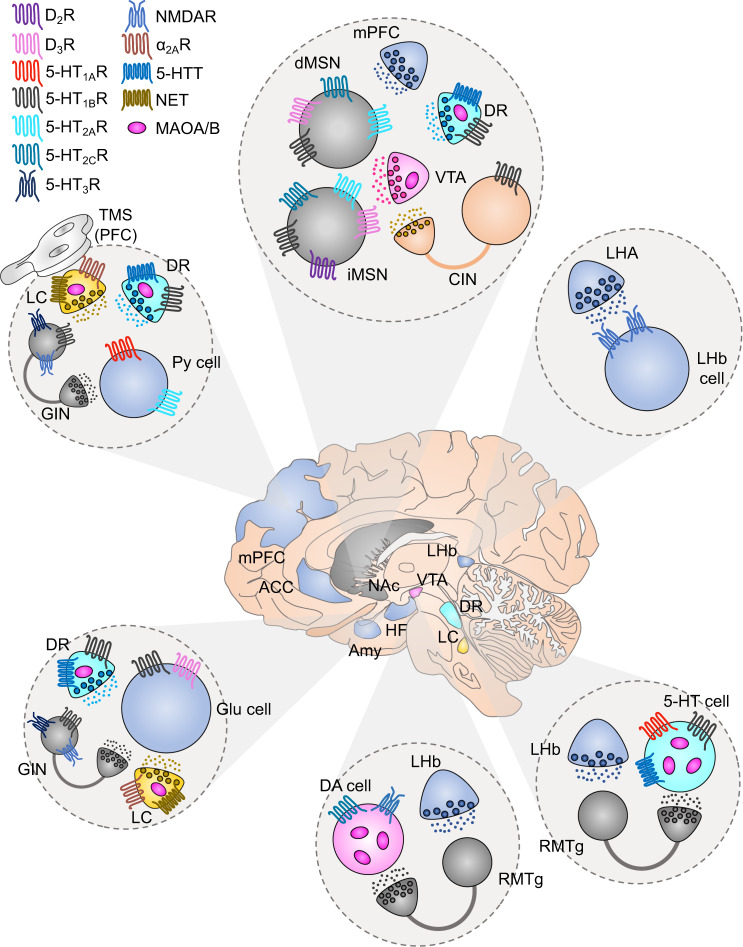
Receptors that are targeted by established treatments in brain regions involved in MDD. In the center, it is depicted the human brain together with different brain areas that are involved in the symptomatology of MDD. Each circle panel shows the neuronal types that have been found to affect depressive-like behavior together with the receptors that they express. GABA neurons/terminals: gray, glutamate neurons/terminals: blue, DA neurons/terminals: pink, 5-HT neurons/terminals: cyan, NE neurons/terminals: yellow, cholinergic neurons/terminals: orange. VTA ventral tegmental area, mPFC medial prefrontal cortex, LHA lateral hypothalamic area, NAc nucleus accumbens, DR dorsal raphe, LHb lateral habenula, RMTg rostromedial tegmentum, ACC anterior cingulate cortex, BA25 Brodmann area 25, HF hippocampal formation, Amy amygdala, dMSN direct medium spiny neuron, iMSN indirect medium spiny neuron, CIN cholinergic interneuron, GIN GABAergic interneuron, Py pyramidal, Glu glutamate, DA dopamine, 5-HT serotonin, 5-HTT serotonin transporter, NET norepinephrine transporter, MAOA/B monoamine oxidase A/B, TMS transcranial magnetic stimulation.

There is a vast body of data implicating certain brain structures in MDD. Post-mortem analyses and MRI studies have reported morphological changes in MDD patients, both in neurons and glia, in several subcortical and cortical brain regions including the hippocampus/subiculum, amygdala, nucleus accumbens (NAc), and prefrontal cortex (PFC) (Fig. 1) [2, 3]. The decrease in hippocampal volume is directly proportional to the number and duration of depressive episodes, especially in early-onset MDD [4, 5]. This volume reduction seems to be related to prolonged increased levels of cortisol, which may relate to the fact that the hippocampus is the brain region with the highest levels of glucocorticoid receptors [6]. Accordingly, numerous studies, not least in animal models, have shown that chronic administration of glucocorticoids leads to changes in mood and cognition [6]. Exposure to prolonged stress and/or stressful events can lead to persistently increased responsivity of the HPA axis. [6]. The activity of the HPA axis is fundamental to the control of several body functions, including metabolism, the immune system, and brain functions such as neuronal survival, neurogenesis, sleep regulation, and memory acquisition. The HPA axis activity is governed by different hormones: corticotropin-releasing hormone (CRH) and vasopressin (AVP) from the hypothalamus; adrenocorticotrophic hormone (ACTH) from the anterior pituitary; and glucocorticoids from the adrenal cortex. By acting via the two GPCRs, CRH1 and V1b, increased levels of CRH and AVP will lead to increased ACTH that will, in turn, increase the secretion of glucocorticoids. Glucocorticoids have a negative feedback action on the HPA axis. A significant number of depressive patients show increased levels of glucocorticoids and a lack of feedback suppression of the HPA axis by glucocorticoids. Antagonism of nuclear glucocorticoid receptors, particularly by mifepristone, has been evaluated in psychotic depression [7]. Likewise, antagonists at the, CRH1 and V1b receptors have been developed as putative antidepressants [8, 9]. In depressive patients, memory formation is skewed towards negative events; this is known as negative bias. Negative bias is characterized by an enhanced focus on negative stimulus, enhanced attention towards potentially threatening stimuli, and the attribution of negative emotional value to environmental stimuli that are considered to have neutral valance by healthy individuals [10]. Negative bias-related memories does not only involve the hippocampus, but also amygdala, anterior cingulate cortex (ACC), PFC, and ventral striatum (i.e. NAc).

There is also a lot of evidence of volume reductions of cortical areas in MDD which correlates to disease progression and therapy responsivity [4] In particular, structural MRI has shown volume reductions in medial PFC and ACC [4]. Transmagnetic stimulation (TMS), which is gaining acceptance as an antidepressant therapy, is often applied to these frontal cortical regions [11]. It has been observed that some frontal cortical regions (including the subgenual cingulate cortex or Brodmann area 25) are metabolically overactive in treatment-resistant depression [12]. Accordingly, this area was targeted with deep brain stimulation (DBS), which is a method where high frequency electrical stimulation is transmitted to specific CNS target areas through an implanted brain electrode [12]. This resulted in a striking and sustained remission of depression in some previously treatment-resistant patients in an open study [12]. However, there is no published randomized clinical trial (RCT) to confirm this antidepressant effect. It seems that patient selection based on multiple parameters, including diffusion tensor imaging and tractography identification of circuitries, and optimized protocols are necessary to improve response and clinical utility of DBS in MDD [13].

Altered excitatory neurotransmission from hippocampus/subiculum and frontal cortex alters the activity of outputs regions. There is accumulating evidence that NAc plays an important role in integrating excitatory neurotransmission from hippocampus/subiculum and frontal cortex to mediate emotionally dysregulated behaviors in MDD [14]. Deficit excitatory inputs to the NAc has been reported to result in reduced activity and overall volume in MDD patients [15] Likewise, repeated stress causes a volume reduction in the NAc of rodents [16] An observational study showed an antidepressant effect of DBS in the NAs [17], but a subsequent RCT failed to replicate this finding [18].

Technological advances in anatomical and functional dissection of neural circuits in animals have identified brain structures that actively regulate mood. Using optogenetics, it has also been described that the reward-brain state is mediated by the feedback loop between the PFC, NAc, and thalamus [19]. One of the major pathways that have been repeatedly shown to display antidepressant-like properties, is the ventral tegmental area (VTA)- NAc dopaminergic (DA) pathway [19, 20]. Phasic optogenetic activation of this pathway elicits rapid antidepressant effects [21]. In accordance, optogenetic activation of the VTA innervating GABAergic neurons located in rostromedial tegmental nucleus (RMTg), disrupts reward consumption [22, 23]. Another region that has gained a lot of attention due to its strong relationship with aversion and depressive-like states, is the lateral habenula (LHb) [24–27]. This brain region stimulates RMTg which in turn inhibits VTA DA neurons [28, 29]. It has been shown that optogenetic activation of excitatory LHb afferents from lateral hypothalamic area or LHb neurons themselves, produces a robust avoidance behavior [30]. Accordingly, DBS of the LHb can reverse depressive-like behaviors in rodents [25], reinforcing a role of the LHb in depression. Furthermore, optogenetic targeting of projections from medial PFC to dorsal raphe nucleus (DRN) will control mobility in the forced swim test, while targeting projections from basolateral amygdala to NAc produces appetitive or aversive response, depending on the type of stimulus [31].

## Neurotransmitters involved in MDD

The current antidepressant medications are targeting a variety of neurotransmitter systems in the brain. The main five classes of antidepressant include selective serotonin (5-HT) reuptake inhibitors (SSRIs), serotonin-norepinephrine (NE) reuptake inhibitors (SNRIs), tricyclic antidepressants (TCAs), monoamine oxidase inhibitors (MAOIs), and atypicals [32]. The first four classes are indirectly enhancing monoamine neurotransmitters, while the latter mainly act as monoamine receptor ligands [32]. Due to the uprising of ketamine which modulates glutamatergic neurotransmission as fast-acting antidepressant, we will divide the antidepressant drugs according to the neurotransmitter system that they affect (monoamine-based versus glutamate-based) [32].

As detailed below, the monoaminergic nuclei, VTA (DA), DRN (5-HT), and locus coeruleus (LC) (NE), send widespread projections to the hippocampus, amygdala, ACC, PFC, and NAc and modulate their functions [33]. A critical role of monoamines in depression was discovered more than 60 years ago when it was found that iproniazid, a drug developed against tuberculosis, was found to exhibit antidepressant properties via inhibition of MAO [34]. During the same period, the putative antipsychotic agent, imipramine, showed antidepressant properties and was approved as the first tricyclic drug against depression [35]. Pioneering work showed that imipramine could counteract NE uptake into presynaptic neurons [36]. It was later found that the antidepressant desipramine, a metabolite of imipramine, is a selective NE reuptake inhibitor. This finding, together with data showing lowered activity of NEergic neurons in depression, led to the catecholamine hypothesis of depression [37]. NEergic neurons from the LC innervate mainly the PFC, amygdala, hippocampus, and hypothalamus [38, 39]. It was subsequently found that several tricyclic drugs for depression also inhibit 5-HT reuptake, and a 5-HT hypothesis of depression was conceived [40]. Direct in situ detection of 5-HT and its rate-limiting enzyme, tryptophan hydroxylase 2, have demonstrated that 5-HTergic terminals are enriched in PFC, hippocampus, amygdala, hypothalamus and basal ganglia output structures [38, 41, 42]. These axonal fields stem from the DRN [42]. Currently, agents that selectively inhibit the reuptake of either NE (e.g. reboxetine) or 5-HT (e.g. fluoxetine, paroxetine, sertraline, and citalopram), or both (e.g. venlafaxine and duloxetine) are commonly used as drugs for depression and anxiety [43]. Bupropion, a NE and DA reuptake inhibitor, is an antidepressant drug with energizing and mood-elevating properties acting on the mesolimbic VTA-NAc and VTA-PFC DA pathways [43].

It has been established that the mesolimbic VTA-NAc connection is a crucial regulator of mood [20] Dopaminoceptive cells in the dorsal as well as ventral striatum (i.e. NAc) are so-called medium spiny neurons (MSNs) and release GABA as a neurotransmitter [44]. In the dorsal striatum, which receives dopaminergic input from substantia nigra and critically regulates locomotion, MSNs are well-divided in two equally large populations named direct medium spiny neurons (dMSNs) or indirect medium spiny neurons (iMSNs) [44]. dMSNs express high levels of DA D_1_ receptors, whereas iMSNs express D_2_ receptors, respectively [45]. The dMSN-iMSN principle is maintained in the NAc, but there is a significant portion of MSNs that express both D_1_ and D_2_ or D_3_ receptors [46–48]. These cells constitute a morphologically distinct MSN type which does not follow the classical dogma of striatal direct and indirect pathway [48]. Nevertheless, selective dMSN and iMSN activation results in opposing functions and show antidepressant and depressive-like properties, respectively [49]. Moreover, recent evidence has shown that both dMSNs and iMSNs encode reward and aversion but with differential action plans [50].

Over the recent years, the understanding of the pathophysiology of MDD has undergone a conceptual shift from monoamine-based models focused on monoaminergic synaptic neurotransmission in localized brain regions; to more dynamic disease models of altered synaptic and neural plasticity in neuronal brain circuitry with a broad focus on not only monoamines but also the excitatory and inhibitory neurotransmitters, glutamate and GABA [51] While dysregulated monoamine neurotransmission may be sufficient to cause depression it has become evident that it is not a necessary condition; converging evidence indicates that changes in glutamatergic signaling, neuropeptides, neurotrophic factors, and brain plasticity play important roles. There is recent data that there is lower synaptic density, measured by a SV2A PET ligand, in hippocampal and cortical networks of MDD patients [52]. In this context, it is interesting to note that tianeptine, an atypical drug for depression, promotes synaptic plasticity by potentiating glutamate AMPA receptor function [53]. Moreover, it has also recently been demonstrated that tianeptine acts as a μ opioid receptor (μOR) agonist to exert antidepressant actions [54].

Studies using magnetic resonance spectroscopy to measure the concentration of intra- and extracellular glutamate, glutamine, and GABA have shown changes in the glutamate system (for example, reduced levels of glutamine, the precursor of glutamate) in MDD patients [55–57]. The changes in glutamate levels in MDD have been reported to show regional differences [58]. A pharmacological link between glutamatergic neurotransmission and MDD was actually found more than 60 years ago when it was reported that D-cycloserine ameliorated depressive symptomatology [59]. It was later found that this effect may be due to partial agonism at the glycine recognition site of the glutamatergic NMDA receptors. As discussed below, several RCTs with the NMDA antagonist, ketamine, have been carried out in the past decade demonstrating a rapid antidepressant effect in MDD patients resistant to monoamine-based antidepressants [60].

## GPCR-mediated effects of approved monoamine-based antidepressants

The eukaryotic repertoire for cell communication and signal transduction is largely mediated by several different receptor families with distinct mechanisms of activation and action. Members from the different receptor families have been implicated in MDD. They range from nuclear receptors, such as the glucocorticoid receptor, ligand-gated ion channels, such as the NMDA receptor, receptor tyrosine kinases, such as BDNF/TrkB, to GPCRs. GPCRs span over 800 members in the human genome and can be roughly divided into two groups; olfactory and nonolfactory [61]. Structurally, GPCRs are composed of a common foundation spanning seven helical transmembrane (TM) domains, with highly heterogenous amino and carboxyl termini [62]. GPCRs can be divided into six classes (A-F) with two newer additions accounting for taste and other receptors [63]. These groups can be further separated based on their pharmacological classification, such as if they bind nucleotides, lipids, amino acids, or biogenic amines. Importantly, they are typically expressed on the cell-surface plasma membrane. Upon activation, GPCRs undergo a conformational change typically involving large outward movements of TM 5 and 6 cytoplasmic tails. GPCRs act as guanosine nucleotide exchange factors which, upon activation, favor the switch of GDP for GTP on the Gα of the inactive Gαβγ heterotrimer [64]. The GTP bound complex undergoes rapid disassociation followed by separation of the Gα and the Gβγ dimer of the Gαβγ complex [65–67]. The Gβγ subunits can then directly interact with several signaling molecules such as G-protein inward rectifying potassium channels (GIRKs) and Ca^2+^ channels. Depending on the Gα that is recruited, various downstream signaling processes may occur [66, 68]. A plethora of molecular pharmacology and careful investigation into GPCR: Gα pairing has revealed that many receptors can signal via several Gα’s depending on variables such as ligand and cellular context [61, 69]. The signaling cascade is terminated starting with GPCR phosphorylation by G-protein coupled receptor kinases which potentiates the recruitment of β-arrestins. β-arrestins sterically block further Gα interactions, and typically internalize the receptor, either recycling it back to the surface plasma membrane or forward the receptor for degradation [70].

GPCRs are appealing for pharmacological intervention and correspondingly serve as the targets for around 1/3rd of all FDA approved medications [71–73], due to several reasons; First, GPCRs act as signal transducers by typically binding an extracellular signal molecule, and respond by relaying the signal through complex cascades to the cell interior. This provides a therapeutic potential in altering the internal state of a target cell group without requiring membrane permeability of the molecule. Second, GPCRs are known to control or influence nearly every relevant physiological process from vision to vasoconstriction, which permits for direct pharmacological modulation of a specific, disease relevant process. Third, GPCRs are known to be activated by a myriad of different ligands, ranging from photons to peptides, which allows for a substantial chemical space in which to design therapeutic molecules to act on these receptors. Using X-ray crystallography or cryo-EM, recent breakthroughs have resulted in atomic resolution structures for more than 70 GPCRs in complex with ligands and intracellular effectors. This enables the identification of binding pockets and performance in silico library screening of small molecules towards GPCRs, providing faster and larger avenues towards drug discovery [74–76].

Monoaminergic neurotransmission is largely based on GPCR signaling. Since the major antidepressant classes affect the monoaminergic systems, they exert their actions either directly or indirectly through GPCRs. There is a delayed onset of therapeutic efficacy of monoamine-based antidepressants and the mechanism(s) underlying their antidepressant actions remain to be fully understood. There is evidence that the late onset therapeutic effects may be the outcome of transcriptional alterations in genes encoding proteins involved in synapse formation or neurogenesis [20, 77]. There is also data suggesting an important role of altered levels and posttranslational modifications of proteins regulating synaptic plasticity and neuronal firing [78]. There are 14 5-HT receptors (13 which are GPCRs), 6 NE receptors and 5 DA receptors. 5-HT_1A_ receptors are critical in regulating 5-HT neurotransmission. The 5-HT_1A_ somatodendritic autoreceptor, which negatively controls 5-HT release, is an important target, since its blockade augments extracellular 5-HT levels [43], whereas postsynaptic 5-HT_1A_ receptors exert behavioral and neurogenic effects of fluoxetine [77]. Accordingly, vilazodone is a combined 5-HT_1A_ partial agonist and an SSRI, and is used for the treatment of depression [43]. Another compound that both inhibits 5-HT reuptake and targets 5-HT receptors is vortioxetine, which binds to 5-HT_1A_ (agonist, postsynaptic receptor selectivity), 5-HT_1B_ (partial agonist), and 5 HT_7_ (antagonist) receptors along with ionotropic 5-HT_3_ (antagonist) receptors. In addition to these 5-HT receptors, antagonism at 5-HT_2C_ receptors plays an important role in the antidepressant actions of mirtazapine and agomelatine [43, 79]. Agomelatine combines 5-HT_2C_ receptor antagonism with agonism at another GPCR, the melatonin MT_1_ receptor and has antidepressant properties, along with beneficial effects on sleep, due to the restoration of circadian rhythmicity. Mirtazapine is also a NEergic α_2A_ autoreceptor antagonist and enhances fronto-cortical NEergic transmission to elevate mood.

Several studies have reported that DA D_2/3_ agonists, some of which are used in the treatment of Parkinson’s disease [80], exert antidepressant actions [81].

## Receptor-mediated actions of glutamate-based antidepressants

In contrast to monoaminergic drugs, some compounds acting via glutamate receptors possess rapid antidepressant effects. It has been reported that glutamate-reducing agents, such as riluzole and lamotrigine, exert antidepressant actions by lowering extracellular levels of glutamate [82–84]. Moreover, it has been established that blockade of NMDA receptors by a single injection of the negative allosteric modulator (NAM) ketamine causes a rapid (within hours) and long (weeks) antidepressant effect [57, 60, 85]. This is a breakthrough in the field, but the psychotomimetic, anaesthetic, amnestic, and addictive properties of ketamine preclude usage on a larger scale. Intense research is focused on finding alternative ways of interfering with glutamate neurotransmission to achieve fast antidepressant actions without severe side effects.

Mapping of pathophysiological processes involving NMDAR is necessary to fully elucidate effects of ketamine. Due to the widespread expression of NMDAR throughout the CNS, the neuroanatomical target(s) and circiutries that mediates ketamine’s antidepressant effects is still poorly understood. Nevertheless, the ability of ketamine to block the firing of LHb glutamatergic projection neurons [86, 87] and GABAergic prefrontal cortical interneurons [88–90], appears important for its antidepressant properties. Several studies have been examining the role of different NMDA receptor subunits in antidepressant responses. NMDAR function is dependent on the subunit composition of these receptors, which are heteromeric assemblies of at least one NR_1_- and other NR_2_- (NR_2A_-_D_) subunits. The functional and pharmacological properties of NMDAR are determined by their NR_2_ subunits and studies have examined the role of the individual NR_2_ subunits in relation to depression-like states. NR_2A_- and NR_2B_-containing NMDARs are the predominant complexes in forebrain regions mediating emotional behaviors. Antagonists specific to the GluN_2B_-subunit are available and have shown that blockade of GluN_2B_ is sufficient to induce antidepressant-like effects of NMDAR antagonists in rodents [88, 91, 92]. However, at the same time, selective deletion of GluN_2B_ subunit in somatostatin positive GABA interneurons in mPFC abolishes the rapid antidepressant actions of ketamine [89]. It is important to remember that no human clinical studies have, so far, replicated the full spectrum of robust, rapid, and sustained antidepressant actions observed with ketamine using alternative drugs that directly inhibit NMDAR function [93].

Even though there are several studies which provide evidence that ketamine’s antidepressant properties stem from its ability to antagonize NMDARs, there is growing evidence supporting that ketamine’s NMDAR-independent actions may also play a role in alleviating depressive symptoms. Preclinical studies indicate that the ketamine metabolite (*2R*,*6R*)-hydroxynorketamine ((*2R*,*6R*)-HNK) exerts antidepressant action with fewer side effects than ketamine by potentiating AMPA receptors [94]. This study reports that ketamine’s NMDAR-dependent actions produce the side effects of the drug, such as abuse liability demonstrated by self-administration, psychotomimetic actions in the prepulse inihibition paradigm and motor icoordination, while the (*2R*,*6R*)-HNK-induced AMPAR activation is responsible for the antidepressant actions [94]. Recent data indicate that metabotropic glutamate receptors are also involved in antidepressant actions of ketamine and (*2R*,*6R*)-HNK. In particular, combined subeffective doses of an mGlu_2/3_ receptor antagonist and (*2R*,*6R*)-HNK or ketamine synergistically exerted antidepressant-relevant actions [95, 96]. These studies illustrates that targeting metabotropic glutamate receptors may achieve antidepressant effects. The evidence for depression treatment via modulation at mGluR’s has actually been known for nearly two decades (for a thorough earlier review on the topic, refer to ref. [97]). There are eight metabotropic glutamate receptors and ligands acting at metabotropic glutamatergic 2, 3, or 5 receptors have shown antidepressant actions and allosteric modulators have entered clinical trials in MDD patients (see below).

## Current GPCR targets, new avenues

### Biased ligands

**Fig. 2:**
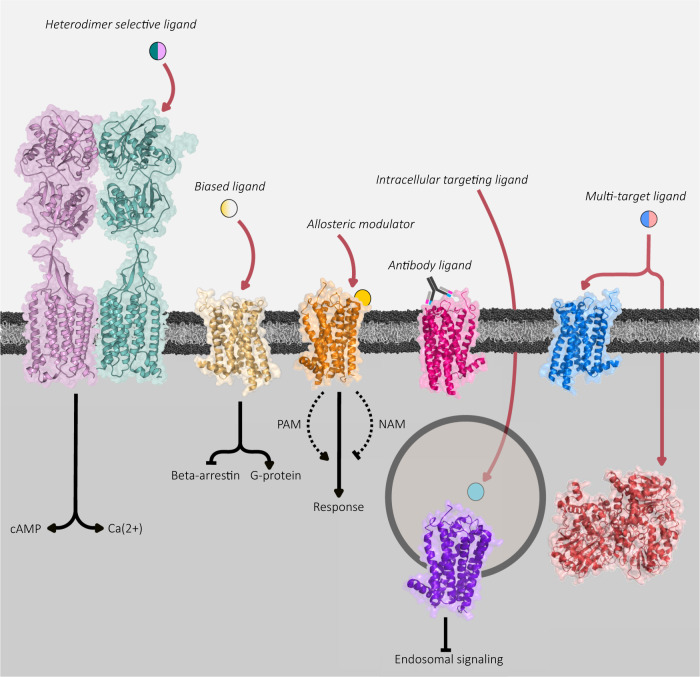
Novel avenues of receptor targeting. Starting from left: A ligand which recognizes a specific dimer pair of receptors with a GABA_B_ receptor as an example. Here, the extracellular region of GABA_B1_ binds the ligand and the second (GABA_B2_) transmembrane receptor recruits G-proteins for downstream signaling cascades. Biased ligands can be used to favor signaling along one signaling pathway while avoiding the other. Similarly, allosteric modulators may be used to regulate the endogenous agonist response, either in an excitatory (PAM) or inhibitory (NAM) fashion. Likewise, antibody-based ligands have been demonstrated both in nature and in recent pharmacological screens to exert excellent target specificity. Specific receptor populations may also be targeted by focusing on intracellular targets such as with the neurokinin-1 receptor. Lastly, the development of rationally designed molecules targeting both a GPCR and an intracellular enzyme may modulate two distinct cellular processes inducing a greater therapeutic response form the drug.

A dominating approach in modern drug discovery concerning ligand development for a target receptor is to selectively modulate, or fine tune its transduction mechanism, favoring one signaling pathway over another (see Fig. 2) [98]. This favoritism is often referred to as signaling bias or functional selectivity [99] (for more terms, see to [100]). The fundamental idea is that as many GPCRs are capable of coupling to different Gα subunits (and thus eliciting different cellular responses), different ligand profiles can be used to favor stabilizing receptor conformations for coupling to a specific intracellular mediator. The concept is not limited to G-protein:GPCR selectivity, but also applies to the degree of G-protein vs β-arrestin recruitment which a ligand may exhibit. This adds complexity to the historical model in which all agonists were thought to stabilize one receptor state, eliciting a signal via one concise pathway [100].

These concepts are valuable consequences for not only rational drug design, but furthermore expands our understanding of GPCRs, discounting them as binary switches and recognizes them as machineries capable of varied, ligand specific conformational changes resulting in a diverse set of outcomes [101]. These expanded outcomes also help us to understand the effects of already established pharmacological treatments on otherwise well characterized receptors.

The goal for any therapeutic agent would be to maximize its positive effects while mitigating negative side effects. Traditionally, the majority of observable side effects have been largely credited to unspecific interactions, resulting in the therapeutic agent activating either unwanted targets, or the target receptor in unwanted cell populations [102]. With the onset of signaling bias, we can now also expand the repertoire of an ideal compound from functional receptor and cell population selectivity, to maximize its properties along a specific signal transduction pathway. Owing to the current advances in structural biology and cell signaling investigation techniques, the search and development of functionally selective ligands has become increasingly popular [103]. Perhaps the most well-known example is the µOR, the main GPCR for morphine (from which the receptor derives its name [104]). Earlier studies suggested that the analgesic effects of the µOR can largely be ascribed to its potential to recruit and signal through Gα_i_ proteins, whereas negative and potentially lethal side effects such as respiratory depression can be attributed to its β-Arrestin-2 signaling [105–107]. Critically, it needs to be mentioned that this phenomenon has also recently been revisited with challenging results [108, 109]. However, the development of µOR agonists which would selectively signal through G-proteins and avoid β-arrestin-2 recruitment garnered clinical interest [110] and the first biased ligand (Oliceridine) was approved by the FDA in 2020.

Specificity has been a major hurdle for the development of pharmacological agents to target psychiatric disorders such as MDD, as the majority of conventionally available therapeutic targets recognize biogenic amines [111, 112]. The aminergic receptors unsurprisingly often share significant homology and conserved orthosteric binding pockets, a key culprit in the resulting amplified difficulty of developing specific, therapeutically feasible molecules [111]. Combining the complex task of designing a receptor selective ligand with that of a pathway specific consequence is by no means an easy task, but one that must be tackled to provide highly beneficial treatment options for depressive disorders. However, this combination of complexities also offers pharmacological versatility as receptors couple to specific intracellular signal transducers within specific cell populations [113, 114]. Ideally, this would imply that providing a molecule encouraging a specific conformational change leading to a precise second messenger coupling would improve the cell and region-specific targeting of the compound. Additionally, the conformational change underlying G-protein recruitment also alters GPCR ligand affinity; in an elegant study using nanobodies which could lock the β2‐adrenoreceptor either in a G-protein bound or an unbound state, the authors characterized the affinity of various compounds for the two receptor states [115]. Remarkably, it was also possible to derive compounds that could discriminate between the two states by over four orders of magnitude and highly G-protein biased compounds could be distinguished [115]. Utilizing such G-protein nanobody mimetics to identify compounds with specificity not only towards G-protein coupling, but also receptor states and perhaps β-arrestin could be highly advantageous for limiting unintended effects of potential new therapeutics and increasing the accuracy of targeting specific cell populations.

The 5HT_1A_ receptor has been well elucidated to serve as potential targets of biased ligands (for an in-depth review, see ref. [114]). The 5HT_1A_ receptor is known to exist in two main populations, as autoreceptors in the raphe nuclei, and as postsynaptic heteroreceptors in the medial septum, PFC, and hippocampus. In the dorsal raphe, G_αi3_ coupling has been shown, whereas G_αo_, G_αi3,_ G_αi1,_ G_αz_ coupling has been reported in the hippocampus and cortex [116, 117]. Balanced targeting of 5HT_1A_ is undesirable, as presynaptic activation inhibits 5-HT release whereas the postsynaptic receptor activation induces synaptic plasticity and neurogenesis [114]. Selective targeting of the postsynaptic heteroreceptor, 5HT_1A_ population could thus provide an efficient therapeutic treatment for depression. As a result, a few selective 5HT_1A_ agonists displaying ligand bias have been developed; F-15599 and F-13714 display ligand bias towards the postsynaptic heteroreceptors and presynaptic autoreceptors, respectively [118–120]. Although this phenomenon is one of regional bias, the authors indeed suggest that it is a direct result of separate affinities for different receptor confirmations (thus G-proteins) and the availability of population specific G-protein subtypes. Interestingly, recent in vivo studies show that, F-15599 improves pattern separation performance, exerts potent antidepressant-like effects and displays a low partiality to induce serotonergic syndrome behaviors [119, 121]. Similarly, another 5HT_1A_ ligand (HBK-17) with a preference for β-Arrestin signaling was recently reported to induce anxiolytic- and antidepressant-like behaviors in vivo [122].

Structure-based methods are invaluable to understand and leverage the key molecular components that underlie ligand–receptor relationships. Around 70 unique GPCR structures have been elucidated, a remarkable worldwide effort since the first high-resolution structure of rhodopsin solved some two decades ago [123]. Although that still leaves some 300 receptor structures to be deciphered, the existing data can and is intensely used to identify residues fundamental for receptor–ligand interactions, micro-switches, and novel binding sites not just in the solved receptor, but in related GPCRs as well. Significant progress has been done on understanding the functional selectivity of the 5HT_2A,_ 5HT_2B_, and 5HT_2C_ receptors [124–127].

On their own, selective targeting of these receptors also holds promise; increasing evidence points to the potential of treating MDD with 5HT_2A_ agonists such as psilocybin, the active ingredient in ‘magic mushrooms’ [128]. Accordingly, FDA has granted a breakthrough therapy designation towards psilocybin-assisted therapy for MDD, with earlier phase II clinical trials indicating encouraging results [129–131]. Although the mechanism behind this phenomenon is still under debate, we have recently gained some structural understanding of 5HT_2A_ antagonism exerted by the second-generation antipsychotics risperidone and zotepine [124]. The structure of 5HT_2A_ displays a conserved hydrophobic cleft surrounding the orthosteric binding pocket composed of key residues such as Ile^3.40^, and Phe^6.44^ and Trp^6.48^ (Ballesteros–Weinstein numbering) of the P-I-F trigger motif which displayed significantly reduced activities when mutated. Interestingly, this same cleft is observed in an inverse agonist structures of the related 5HT_2C_ receptor, but not in its agonist bound state. This helps to highlight the importance of ligand interactions with these residues to promote the inactive conformation, which should be taken into consideration when designing potential new agonists at 5HT_2_ receptors. Additionally, the structure reveals a side-extended cavity connecting the plasma membrane with the orthosteric binding pocket with less conserved hydrophobic residues including a Gly^5.42^, a unique structural detail shared only between 5HT_2_ receptors. Both risperidone and zotepine maintain the highly conserved aminergic salt-bridge with Asp^3.32^, and the binding of risperidone also relies on a more unusual residue, Val^7.39^. Intriguingly, the same is observed also with the parent molecule of risperidone, in the ritanserin bound structure of the 5HT_2C_ receptor. Recently, the structures of LSD bound 5HT_2A_ and more strikingly, the 25-CN-NBOH bound 5HT_2A_ -G_αq_ structures were also elucidated [132]. Once again, large outward shifts of TM5 and 6 are seen, while in the 5HT_2A_ -G_αq_ structure, a smaller inward shift of TM7 is observed. Trp^6.48^ is observed to make hydrophobic contact with 25-CN-NBOH coinciding with the TM6 outward movement and the displacement of Phe^6.44^ of the P-I-F trigger motif. Several critical residues for G_αq_ signaling such as Arg^ICL2^ and Asn^2.37^ were discovered, and Asn^6.55^ was confirmed to be essential for 5-HT but not LSD potency at the receptor. Excitingly, the authors were also able to compare the calculated size of ligand binding pockets, and found that each ligand compared showed significant differences, with the G_αq_ bound state being significantly more open on the extracellular side in comparison with LSD. Most likely, this phenomenon is ligand specific, however the clarification of more ligand-bound G_αq_ coupled structures are necessary to solidify the observation.

Indeed, further work on understanding the functional selectivity of the 5HT_2C_ receptor yielded similar interesting observations [126]. Here, Peng et al. solved the receptor structure in both an agonist (ergotamine) and an inverse agonist (ritanserin) bound state which allowed them to investigate the structural aspects of ligand promiscuity and selectivity in 5HT_2_ receptors. Similarly to 5HT_2B_ and other biogenic amine receptors, 5HT_2C_ activation included a significant outward movement of the intracellular TM6, and a shift of the Trp^6.48^ P-I-F motif micro-switch. Excitingly, they identified the conserved residues Trp^6.48^ and Phe^6.44^ to be largely involved in G-protein coupling, without any influence on arrestin activity. The structure also suggests two residues mentioned with the 5HT_2A_ inactive structure (Gly^5.42^ and Val^7.39^) to mediate ritanserin’s selectivity for the 5HT_2_ receptors.

Apart from key residues responsible for ligand–receptor specificity, information concerning critical switches and residues in 5-HT receptors mediating subsequent signal bias are crucial. Structural data from the Roth lab suggests that the preferential β-arrestin2 recruitment of ergoline compounds such as LSD on the 5HT_2B_ receptor is both a result of a slow disassociation of the compound due to recruitment by the second extracellular loop (ECL2) and a ligand recognition motif in the extended binding pocket, particularly with the seventh transmembrane residue Leu^7.35^ [127]. Similar data for the importance of this residue as a factor in signal bias has been indicated for other aminergic receptors such as the κ-opioid receptor and the β_2_-adrenergic receptor [133, 134]. Likewise, the phenomenon of the lid-like ECL2 interactions increasing ligand residence time resulting in augmented β-arrestin recruitment has been suggested in other aminergic receptors [125, 135]. Intriguingly, residue contacts on the orthosteric binding pocket of 5HT_2B_ failed to show any bias towards Gα_q_ signaling or β-arrestin2 recruitment, instead the key residues (Thr^3.37^, Ala^5.46^) contributed uniformly towards their recruitment. These observations imply that coordinated, receptor wide changes contribute to the degree of β-arrestin2 recruitment with specific residues contributing on the preference of G-protein or β-arrestin2 recruitment [136]. For example, in the LSD-5HT_2A_ structure, mutation of Ser^5.46^ did not change binding affinity, but did alter disassociation rate of LSD, highlighting the potential role of this residue in β-arrestin recruitment [132]. In the case of the 5HT_2B_ receptor, contact with TM7 Leu^7.35^ appears to be crucial for triggering biased signaling. Lastly, the availability of the 5HT_2B_ structure is also important as it should allow better attempts at avoiding its activation. It is abundantly expressed in heart valves and can thus cause serious side effects for medications that are meant to target 5-HT receptors in brain cell populations [137].

Further meta-analysis of several aminergic GPCRs solved both in the active and inactive state divided the common activation pathway of these receptors into four layers of 34 conserved residue pairs [138] and break the transmembrane helical changes down to three main steps: (1) elimination of TM3-TM6 contacts (2) TM3-TM7 contact formation and (3) repacking of TM5 and 6. The authors then went on to exploit these details and successfully designed several previously unknown constitutively active and inactive mutants for the 5HT_1B_ and 5HT_7_ receptors. (For detailed reviews on structural insights of serotonergic receptors, refer to refs. [136, 139]).

The identification of these key residue sites for ligand interactions that maximize ligand: receptor specificity, stabilize different receptor states and define specific signaling outcomes are crucial for the future design of selective, tailored ligands, especially as increased computational power and access to in silico screening tools provide faster routes of ligand discovery [76].

### Allosteric modulators

The majority of ligands mentioned above exert their actions largely through interactions in the orthosteric binding site of their respective targets, however the potential of allosteric modulators should not be underrepresented. As their names suggest, allosteric modulators can be defined as ligands which bind to an alternative, distinct site separate from the endogenous agonist and thereby potentiate or inhibit the orthosteric bound agonist response (Fig. 2) [140, 141]. Thus, allosteric and orthosteric binding are not mutually exclusive but rather influence each other [142]. Positive allosteric modulators (PAMs) are substances which decrease the disassociation rate of an agonist from the orthosteric site, whereas NAMs increase the disassociation rate. Thus, pharmacologically speaking PAMs increase the apparent affinity of the endogenous agonist for the receptor, while NAMs decrease the ligand affinity. Neutral modulators which compete for the allosteric binding site with PAMs and NAMs without influence over agonist off-rate and affinity have also been described [141, 142]. As these effects are dependent on the orthosteric ligand it also means the observed effects are specific for each pair analyzed, thus especially in the context of modulating an endogenous process, modulators should be interpreted in the presence of the endogenous agonist whenever possible [143].

Allosteric modulators present several pharmacological advantages compared to traditional ligands, especially in the context of neuropharmacological challenges such as depression treatment. With regard to drug specificity, allosteric binding sites are less conserved between receptors compared to their orthosteric counterparts and thus, allosteric modulators may offer greater accuracy with regards to receptor selectivity [141]. Furthermore, a common issue with chronic administration of receptor agonists is receptor desensitization, however PAMs only modulate the effects of an endogenous agonist when it is present and bound to the receptor [141]. Instead of a continuous stimulation of an exogenous agonist which would result in a change in the temporal signaling profile of the target receptor, PAMs may provide therapeutic alternatives with greater long-term reliability.

The potential for allosteric modulators in treating MDD is considerable and can be foreshadowed by some existing medications. For example, the family of benzodiazepines which have been prescribed for anxiety disorders since the 1960s, act as allosteric modulators on the GABA_A_ receptor [144]. Furthermore, ketamine, the previously discussed breakthrough antidepressant treatment, is interestingly an allosteric antagonist of the NDMA receptor, occupying the same allosteric binding site as MK-801 (trade name dizocilpine) [145].

Initial efforts on uncovering the mechanism of glutamate in anxiolytic and antidepressant drugs indicated that mGluR_5_ antagonists display antidepressant properties, partly through an inhibitory crosstalk with NMDA receptors [146, 147]. Although the link between this receptor and MDD remains to be fully understood, there is evidence that the antidepressant action of mGluR_5_ antagonists is mediated by parvalbumin-positive GABAergic cortical interneurons [148]. There are several mGluR_5_ NAMs that have been developed and showed promise in preclinical settings but with rather limited clinical outcomes. AZD2066, an mGluR_5_ NAM underwent an RCT (NCT01145755), but it was prematurely stopped. Basimglurant, also an mGluR_5_ NAM failed to show significant improvement in the primary outcome measures but revealed efficacy among secondary outcome endpoints in an MDD focused RCT [149]. GRN-529, another NAM acting on mGluR_5_ was shown to improve depressive behaviors in vivo in rodents, but remains to be examined in human clinical trials [150].

Antidepressant actions of compounds acting on other mGluR’s, more specifically in groups II and III, have also been elucidated; the mGluR_2/3_ NAM Ro449153 exhibited promise in preclinical settings and another mGluR_2/3_ NAM BCI-632 displayed antidepressant effects in rodent models and has been evaluated by an RCT for MDD treatment [151]. JNJ-40411813, an mGluR_2_ PAM, was evaluated as an adjuvant treatment in depression and anxiety, with no significant primary outcome efficacy scores versus placebo treatment however it showed efficacy at secondary outcome measures of both depression and anxiety [152]. THIIC, a mGluR_2_ PAM, shows significant antidepressant effects in rodent models of depression [153]. Recent work employing transgenic animals and optogenetics indicate that selective inhibition of mGluR_2/3_ in the PFC induces antidepressant effects which was also observed with NAMs for both receptors although with different modes of action; mGluR_2_ via thalamocortical presynaptic glutamate release enhancement and mGluR_3_ via mediating postsynaptic plasticity [154].

### Polypharmacology

Instead of designing a highly specific and tailored ligand for one receptor (i.e. a magic bullet), targeting one cell population and pathway, approaches have been made to pursue several targets with one compound (Fig. 2) [155]. As depression itself exists as a multifaceted disease with a multidimensional clinical profile likely as a result of numerous molecular alterations in the patient, it is unlikely that effective treatment can be achieved by pharmacologically focusing on one target [43, 112, 155]. Therefore, modulating two or more targets is probably necessary for a positive clinical outcome, which can be achieved either by combining multiple drugs (polypharmacy) or designing a drug with multiple targets (polypharmacology). Most classical drugs used to treat depression such as tricyclics display a substantial effect outside of their intended target (biogenic amine transporters) and as mentioned earlier, several already existing antidepressants such as agomelatine, vilazodone, and vortioxetine similarly target several receptors at once [43, 79, 155]. Lastly, the effect of several ‘single action’ SSRIs have been shown to be dependent or modulated by several 5-HT receptors [156–158]_._ Thus, it is clear that the large majority of existing medications already target a plethora of different cellular components. (For a comprehensive review on polypharmacy and major depression treatment see ref. [112]).

Other avenues of multitarget compounds could similarly extend to acting on both a receptor and a non-receptor such as an enzyme, for example monamine oxidase (MAO). MAO inhibitors (incl tranycypromine) are used in atypical forms of depression, and compounds such as 8-(3-Chlorostyryl)caffeine have been found which both acts as an adenosine A_2A_ receptor antagonist and a MAO-B inhibitor [159]. Owing to the availability of the crystal structure of both proteins, large scale virtual docking enabled the discovery of several specific compounds which could act on both targets [160]. Similar approaches have been used to identify novel MAO inhibitors which also act on several other GPCRs such as DA and histamine receptors [161, 162]. As the structures of the 5HT_2A,2B,2C_ and 5HT_1B_ receptors have already been solved, they present an opportunity for discovering dual acting compounds on relevant depression targets. Additionally, computational methods have been successfully exploited to automate the design of ligands with polypharmacological, CNS penetrant profiles [163].

### Receptor dimers

The idea of higher order receptor structures, i.e. dimers, trimers, and oligomers, adds another dimension of GPCR pharmacology. Since their hypothesis in the 1980s, numerous studies have now shown that receptor signaling is not only largely influenced by the conformational changes that take place following direct ligand contact, but that these conformational changes can also be influenced by allosteric interactions with other receptors on the plasma membrane [164–166]. Receptor dimerization on the cell-surface membrane is obligatory to class C members, making them extremely peculiar in terms of pharmacological function and energetics. One of the best documented cases of a functional heterodimer is the class C GPCR GABA_B_ complex, wherein one subunit (receptor) binds the ligand, and the other exerts an intracellular response via G-protein recruitment (Fig. 2) [167]. Outside of Class C dimers, many efforts to discover other dimers have shown promise but the physiological reality of these must be carefully interpreted, as overexpression models and immunoprecipitation methods may highlight mere transient interactions which are not fully relevant or yet understood.

The allosteric influences between receptors can switch the coupling profile of a GPCR and influence maturation and internalization rates [168]. 5HT_1A_ and 5HT_7_ dimers have also been suggested in hippocampal neurons with effects on 5HT_1A_ GIRK activation [169]. The different receptor states (monomers, dimers, oligomers) also display differential affinities for ligands. Separate from altered ligand affinity, heterodimers have been proposed to facilitate cross-talk between neurotransmitter GPCRs (e.g. D_2_-A_2A_ heterodimers) [170], neurotransmitter and neuropeptide GPCRs (e.g. 5HT_1A_-GalR heterodimers) [171], neurotransmitter receptor-neurotrophic factor receptors (e.g. 5HT_1A_-FGFR1 heteromers) [172, 173] and GPCRs and ionotropic receptors (e.g. D_1_-NMDA heteromers) [174]. However, the challenge of evaluating the therapeutic potential of these receptor associations remains difficult, especially in vivo. (for an in depth-review on GPCR heteromerization criteria see [175]).

These associations can be exploited to enhance therapeutic potential by targeting the unique receptor complex. Heteromer targeting drugs may also offer increased precision as they could leverage the receptor species expression heterogeneity amongst different cell types. Two compounds joined by a linker which can recognize and bind the two receptors in a protomer (known as bivalent ligands) have been developed for a different receptor groups and have been shown to be successful in applications such as nociception reduction [176, 177]. Bivalent ligands targeting depression targets are still lacking, however. A large challenge here is that these linked compounds are bulky and are thus difficult to be made blood–brain barrier (BBB) penetrant [178]. Although the literature is scarce concerning receptor dimers in depression, D_1_-D_2_ dimers were shown to be elevated in the striatum of MDD patients, and interference of these dimers in a rats significantly reduced immobility time in the FST [179]. Further potential targets are outlined in [180].

### Antibody-based approaches to target GPCRs

A field undergoing significant growth and development in the last two decades is immunotherapy and biologics based treatments. Immune recruitment approaches in cancer have been revolutionary, reflected by the Nobel Prize in Physiology or Medicine in 2018 being awarded for the development of immune checkpoint therapies. Autoimmune diseases such as multiple sclerosis have seen positive outcomes with antibody-based therapies such as rituximab and inflammatory bowel disease and rheumatoid conditions are currently often treated with anti-inflammatory antibodies.

Traditionally, antibody generation against GPCRs has been notoriously difficult, largely due the difficulty of extracting and preparing them in their native state based on their lipophilic environment. Furthemore, in vivo, only the extracellular portion of the receptor in its native form is available for the antibody to recognize. However, the largest degrees of variance between receptors are seen in the loops, N and C termini, which should theoretically provide some relief over antibody specificity. As most GPCRs display the N terminus and extracellular loops (ECLs) on the antibody accessible extracellular side, N-terminally or ECL targeted antibodies are appealing for certain therapeutic applications. Interestingly, this phenomenon can be observed in nature as antibodies directed against GPCRs have long been described in the context of Graves’ disease [181]. Here, autoantibodies are generated against the thyroid-stimulating hormone receptor and act as an agonist on the receptor stimulating the proliferation of the thyroid gland.

Antibody-based therapeutics may be useful for GPCR targeting, especially for the aminergic receptors, due to their extreme target specificity and potential ability to act as a ligand (Fig. 2). Accordingly, antibody screens for specific antibodies with agonist properties have been described for targets such as GLP-1 for purposes of diabetes treatment [182, 183] and more recently, the chemokine receptor CCR1 [184].

Even if one manages to generate potent GPCR targeting antibodies, the issue of BBB permeability is immediate, especially in the context of MDD treatment. Although the mechanism of uptake remains unknown, autoimmune diseases such as NMDA receptor encephalitis are caused by autoantibodies against a neuronal receptor which are able to cross the BBB [185]. Another similarity in nature is Ophelia’s syndrome, a subset of Hodgkin’s disease which develop autoantibodies against mGluR_5_ [186]. Additionally, chronic stress, anxiety, and depression have all been linked to a more vulnerable and leaky BBB which would be useful from the perspective of antibody therapy against relevant neurological targets [187, 188]. Similarly, antibody engineering techniques such as the development of bispecific antibodies targeting the transferrin receptors have been extensively used to facilitate a greater degree of BBB penetrance [189–191]. Together, the present antibody engineering techniques and disease phenomenon described in nature do not deny the feasibility of BBB penetrant GPCR targeting antibodies.

An extension of modern antibody therapies geared against GPCRs can be observed in the calcitonin receptor like receptor (CLR) treatment of migraines [192]. A fully human antibody named Erenumab was approved by the FDA and EMA since 2018 for pain management in migraines. It acts as an antagonist of the receptor with a reported IC_50_ of 2.3 nM in cell based functional assays [193]. Erenumab is peculiar as it is bispecific, a fusion of two antibodies for the N terminus of CLR and the N-terminal portion of the receptor activity-modifying protein (RAMP)-1. RAMPs are single pass membrane proteins, belonging to the group of adaptor proteins, which themselves are important for the ligand selectivity, trafficking, signaling, and degradation of GPCRs. Although Erenumab is not BBB permeable, it highlights the success and possibility of developing antibody therapies targeting GPCRs, which can be engineered for BBB permeability in MDD treatment applications.

### Intracellular GPCR targeting

The classical model in which GPCR signaling occurs exclusively on the surface plasma membrane has also been expanded on as continued receptor signaling within several intracellular compartments has been elucidated [194]. More specifically, GPCR signaling has been shown to occur on the nuclear, mitochondrial, endosomal, and Golgi membranes in addition to the surface plasma membrane [195–198]. Some receptors such as those localized to early endosomes appear to maintain their intact signaling configuration from the agonist they bound on the surface membrane and then repurpose this signaling once they are internalized. Others, such as cannabinoid 1 and melatonin type 1 receptors on the mitochondrial membrane seem to serve an entirely unique, intracellular purpose [196, 199]. Accordingly, these cascades while largely unexplored hold potential for treatments towards a variety of disease (Fig. 2). One such example is the substance P bound neurokinin 1 receptor which continues to elicit a signaling cascade resulting in pain transmission once internalized in endosomes. Jensen et al. showed that targeting of this endosomal signaling complex using membrane-anchored antagonist conjugates is able to constrain the pain transmission more efficiently than “standard” surface plasma membrane receptor targeting antagonists [197]. Another approach interfering with the same receptor was successfully accomplished using pH sensitive nanoparticles containing an antagonist which were endocytosed into the same compartments as the active neurokinin 1 receptor, releasing the antagonist [200]. Additionally, an appealing receptor in dopaminergic transmission and addiction research, the trace amine associated receptor 1 was also recently shown to couple to different G-proteins depending on the intracellular compartment, thus eliciting different signaling cascades when bound to the psychostimulant amphetamine [201]. While so far only a handful of receptors have been shown to be capable of mediating signal transduction pathways from an intracellular compartment, this emerging field garners great potential for more efficient and specific receptor targeting. Furthermore, research towards intracellular signaling in other pathological states such as MDD where normal cell-surface signaling may be crucial for normal function, but intracellular states may provide better treatment targets.

### Role of adaptor proteins in GPCR-mediated actions with an emphasis on Homer and p11

Adaptor proteins link protein-binding partners together and facilitate the formation of signaling complexes. Many adaptor proteins, including RGS and AGS proteins, regulate GPCR signaling, but here we focus on GPCR-interacting proteins. Such proteins are important for the ligand selectivity, trafficking, signaling, and degradation of GPCRs [202]. Studies on how adaptor proteins modulate GPCRs have led to successful drug development. Receptor activity-modifying proteins (RAMPs) are single pass membrane adaptor proteins which have turned out to be critical for GPCR functions. RAMPs were initially identified by their ability to bind to and determine the pharmacology of the calcitonin receptor-like receptor (CLR). The interaction of RAMPs with GPCRs is of considerable importance for drug discovery. CLR/RAMP1, or CGRP receptor, antagonists have been developed for the treatment of migraine headache. As mentioned above, specific antisera has been developed, but there are also small molecules antagonizing the CGRP receptor in the clinics [203].

Two GPCR-interacting proteins that have implicated in MDD are homer and p11. Homer is an immediate early gene with a PDZ domain which bind to the cytoplasmic tail of mGluR5, and regulate the cell-surface localization and signaling of this GPCR [204]. It has indeed been shown that various antidepressants increase homer1 in PFC [205]. Accordingly, viral overexpression of homer1 in the PFC causes antidepressant effects in rodents [205]. Moreover, it was subsequently shown that intravenous injection of a cell-membrane-permeable TAT-Homer1 construct causes antidepressant effects [206]. In this study, it was also shown that Homer1-mediated enhancement of mGlu5 signaling promotes antidepressant effects by potentiating AMPA receptor activation [206]. Conversely, siRNA-mediated knockdown of homer1 in mPFC enhanced depressive-like behavior [205]. Interestingly, a linkage between Homer and MDD has been described in a genome-wide association study, indicating a possible role for Homer in the pathophysiology of depression [207].

An adaptor protein that is implicated in 5-HT and glutamate signaling is P11 (S100A10, annexin II light chain, calpactin I light chain). P11 is a multifunctional protein of the S100 protein family which forms a heterotetrameric scaffold with AnnexinA2 particularly at cell membranes. P11, alone or together with AnnexinA2, interacts with several ion channels and receptors and regulates their cellular localization and function [208]. p11 levels are downregulated in the cingulate cortex and the ventral striatum from MDD patients and suicide victims [209–211]. Constitutive p11 KO mice show a depression-like behavioral phenotype in several well-established models that measure behavioral despair (e.g., forced swim and tail suspension tests) or anhedonia (e.g., sucrose preference test) [211]. Site-specific conditional knockout of p11 in NAc cholinergic interneurons produces a depression-like behavioral-phenotype [212].

**Fig. 3:**
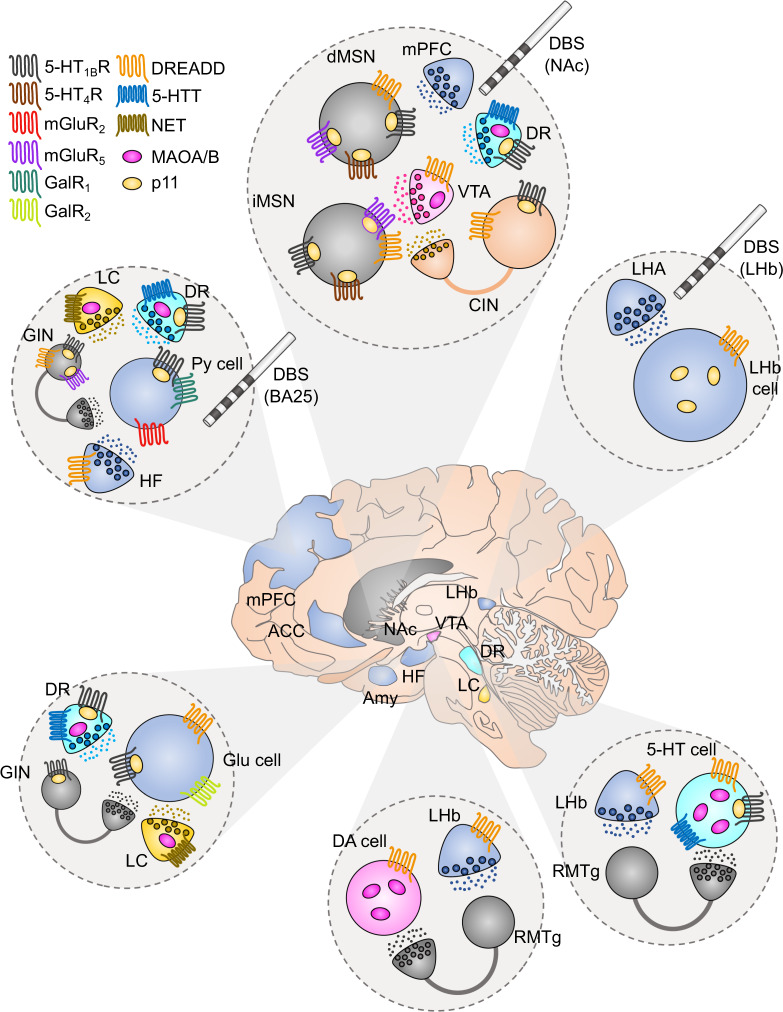
Novel GPCR-based therapeutical strategies in brain regions involved in MDD. In the center, it is depicted the human brain together with different brain areas that are involved in the symptomatology of MDD. Each circle panel shows the neuronal types that have been found to affect depressive-like behavior together with the receptors that they express. GABA neurons/terminals: gray, glutamate neurons/terminals: blue, DA neurons/terminals: pink, 5-HT neurons/terminals: cyan, NE neurons/terminals: yellow, cholinergic neurons/terminals: orange. VTA ventral tegmental area, mPFC medial prefrontal cortex, LHA lateral hypothalamic area, NAc nucleus accumbens, DR dorsal raphe, LHb lateral habenula, RMTg rostromedial tegmentum, ACC anterior cingulate cortex, BA25 Brodmann area 25, HF hippocampal formation, Amy amygdala, dMSN direct medium spiny neuron, iMSN indirect medium spiny neuron, CIN cholinergic interneuron, GIN GABAergic interneuron, Py pyramidal, Glu glutamate, DA dopamine, 5-HT serotonin, 5-HTT serotonin transporter, NET norepinephrine transporter, MAOA/B monoamine oxidase A/B. DREADD designer receptors exclusively activated by designer drugs, DBS deep brain stimulation.

As shown in Fig. 3, p11 is co-localized with 5-HT_1B_ and/or 5-HT_4_ in many cell types of the cerebral cortex, hippocampus, and NAc [211, 213]. The third intracellular loop of 5-HT_1B_, 5-HT_1D_, and 5-HT_4_ interact with p11 [211, 214]. p11 increases the surface expression and signaling via of 5-HT_1B_ and 5-HT_4_ and agonists at 5-HT_1B_ or 5-HT_4_ exert antidepressant action which are dependent on p11 [211, 214]. More recently, it has been shown that stimulation of a 5-HT1B/p11 pathway cholecystokinin (CCK) GABAergic interneurons of the dentate gyrus region of hippocampus is important for initiating the therapeutic response to fluoxetine [215]. In fact, several antidepressant treatments, including fluoxetine, imipramine, tranylcypromine, and electroconvulsive therapy have been shown to up-regulate p11 levels in frontal cortex and hippocampus from rodents [211]. Accordingly, p11 KO mice show reduced responses to antidepressants in various tests for antidepressant efficacy [211]. Likewise, selective deletion of p11 in corticostriatal layer 5a or hilar hippocampal interneurons blunts the antidepressant action of SSRIs [216–218].

Using the chronic unpredictable mild stress model of depression and lentiviral knockdown of hippocampal p11 suggested that p11 may also have a key role in the sustained antidepressant effect of ketamine [219]. A direct link between p11 and the glutamate system is that p11 binds directly to a Ser-Thr-Val sequence in the cytoplasmic tail of mGluR_5_ and facilitates its accumulation at the plasma membrane [148]. p11 overexpression potentiates mGluR_5_ agonist-induced calcium responses in cultured cells [148]. Knockout of mGluR_5_ or p11 specifically in forebrain glutamatergic cells in mice produces depression-like behaviors [148]. On the contrary, deletion of mGluR_5_ or p11 in GABAergic neurons results in antidepressant-like behaviors [148].

It has also been shown show that SMARCA3, a chromatin-remodeling factor, is a target for the p11/annexin A2 heterotetrameric complex [220]. Formation of this complex increases its nuclear localization and the DNA-binding affinity of SMARCA3. SSRI-induced neurogenesis and behavioral responses are abolished by constitutive or mossy cell-specific knockout of SMARCA3 [218, 220]. Apart from annexin A2, it has been reported that p11’s interaction with Ahnak exhibits crucial role in depressive-like behavior [221]. Particularly, p11/Ahnak complex increases the trafficking to the membrane of L-type voltage-gated calcium channels [221].

## Circuitries involved in MDD and targeted via neuropeptide GPCRs

Neuropeptides are short proteins ranging from 3 to around 40 amino acids and very often coexist with classical transmitters in the brain. They act via GPCRs to play a role particularly when the nerve cells are challenged by stress or injury. Thus, neuropeptide receptors are considered as attractive targets for pharmacological intervention of depression. We have in other places of this review indicated roles for GPCRs targeted by CRH, AVP, and endorphins in MDD. In this section, we focus our discussion on receptors of galanin, neuropeptide Y (NPY), and oxytocin.

Galanin is a 29/30 amino-acid neuropeptide with three G-protein coupled receptors termed as GalR_1_, GalR_2_, and GalR_3_ [222]. Many studies have demonstrated that galanin and its receptors are involved in the pathology of depression. Thus, the activation of GalR_1_ and GalR_3_ results in depression-like behaviors, whereas stimulation of GalR_2_ leads to antidepressant-like effects [223]. Accordingly, GalR_2_-knockout mice show depression-like behaviors, while GalR_2_-overexpressing mice exhibit antidepressant-like behaviors [224, 225]. It has been reported that GalR_1_ mRNA is significantly and selectively upregulated in the vPAG of rats exposed to chronic mild stress (CMS) and GalR_1_-siRNA knockdown of the upregulated GalR_1_ reverses depression-like behaviors [226].

NPY exerts its responses via five receptor subtypes, termed Y_1_R, Y_2_R, Y_4_R, Y_5_R, and Y_6_R and has been implicated in anxiety and depression [227, 228]. Among them, Y_1_R mediates NPY-induced antidepressant activity in the olfactory bulbectomized rats (OBX) [229] as well as in the forced swim test in mice [230]. Meanwhile, Y_5_R antagonist has antidepressant-like effects in CMS [231] and FSL [232] rats.

Oxytocin is one of the most important neuroregulators mediating social behaviors and stress-related disorders and plays an antidepressant role in depression [233]. It has been demonstrated that oxytocin levels are inversely correlated with depressive symptoms [234]. Administration of oxytocin were shown to reduce immobility time of FST in mice [235] and reverse depressive-like behaviors and high plasma corticosterone level in postpartum depression rats [236], indicating that central oxytocin exerts antidepressive effects.

## Orphan GPCRs localized in circuitries involved in MDD

**Fig. 4:**
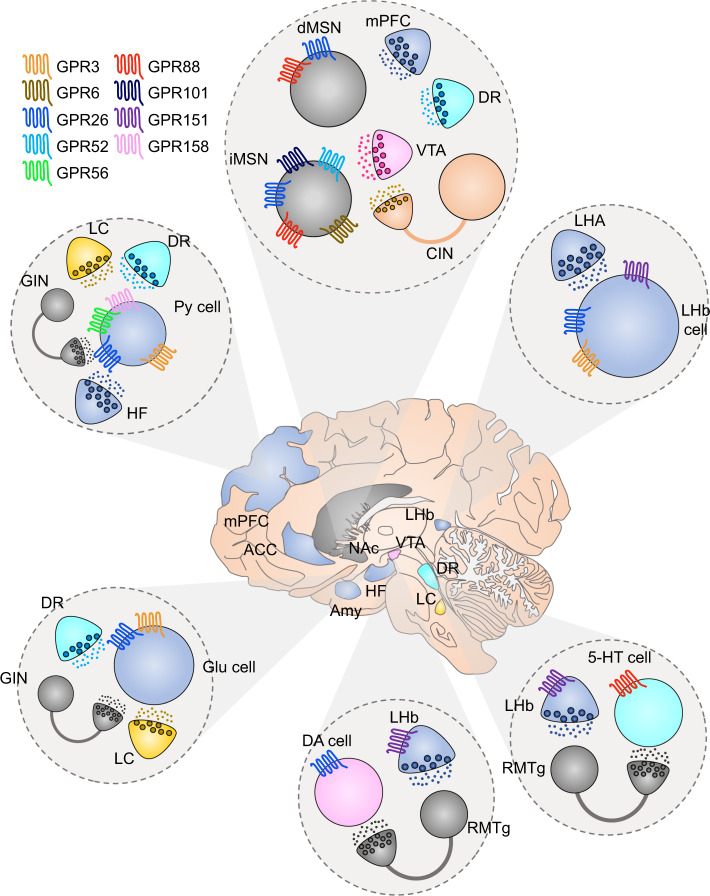
Orphan GPCRs expressed in brain regions involved in MDD. In the center, it is depicted the human brain together with different brain areas that are involved in the symptomatology of MDD. Each circle panel shows the neuronal types that have been found to affect depressive-like behavior together with the receptors that they express. GABA neurons/terminals: gray, glutamate neurons/terminals: blue, DA neurons/terminals: pink, 5-HT neurons/terminals: cyan, NE neurons/terminals: yellow, cholinergic neurons/terminals: orange. VTA ventral tegmental area, mPFC medial prefrontal cortex, LHA lateral hypothalamic area, NAc nucleus accumbens, DR dorsal raphe, LHb lateral habenula, RMTg rostromedial tegmentum, ACC anterior cingulate cortex, HF hippocampal formation, Amy amygdala, dMSN direct medium spiny neuron, iMSN indirect medium spiny neuron, CIN cholinergic interneuron, GIN GABAergic interneuron, Py pyramidal, Glu glutamate, DA dopamine, 5-HT serotonin.

Technological advances in transcriptional profiling of distinct cellular subpopulations, but also in situ detection of mRNA transcripts has revealed expression patterns of novel orphan brain-specific GPCRs (Fig. 4).

As we mentioned above, the activity of the NAc is crucial for the expression of both positive and negative emotions. There are several orphan GPCRs, such as GPR88, GPR101, GPR52, and GPR6, which display a robust striatal expression and have been associated to anxiety and/or depressive-like states [237]. GPR88 mRNA levels have been described to be altered after the treatment with antidepressants in both animal models and humans [238, 239]. GPR88 displays the highest amino-acid sequence homology to the amine-GPCR family and specifically with 5-HT_1D_ receptor [240]. GPR88 is strikingly expressed in the corticostriatal contacts of both dMSNs and iMSNs with some enrichment to striosomes [241, 242]. GPR88 is coupled to Gα_i_ and affects the MSN excitability through impeding the membrane trafficking of AMPAR [241]. Selective of GPR88 deletion in iMSNs is sufficient to reproduce the anxiolytic phenotype observed in the global GPR88 KO mice [243, 244]. There is evidence that GPR88 is expressed in the DRN and regulates 5-HT levels [245, 246]. Although, it has been shown that GPR88 mRNA levels are changed with antidepressant treatment [238, 239] further behavioral studies are needed to evaluate the effect of GPR88 on depression-like behavior. Whereas the endogenous ligand of the receptor remains elusive, several centrally active ligands towards GPR88 has been developed [247]. Another GPCR which has also been characterized by its striatal enrichment and its relationship to adrenergic receptors, is GPR101 [248]. It is a Gα_s_, Gα_q/11_, and Gα_12/13_ coupled receptor and is enriched in the matrix compartment of the striatum [248, 249]. Mice lacking GPR101 display higher immobility time in the forced swim test [248]. GPR52, a Gα_s_ coupled orphan GPCR, is localized uniquely in iMSNs of striatum and GPR52 KO mice demonstrate an anxiety-like phenotype [237, 250]. The recently solved crystal structure of GPR52 is providing new insights for the development of novel pharmacological tools [250]. Future studies are required to examine the role of GPR52 in depression-like behaviors. However, since Gα_i_ coupled D_2_ receptors in iMSNs exert antidepressant properties, it is not unlikely that GPR52 antagonists may be antidepressant. Recently, it has been shown that small molecule GPR52 agonists (BD442618, BD502657) can reduce ropinirole actions. Accordingly, future development of GPR52 antagonist might enhance D2 receptor agonist actions. modulate D_2_ receptors and GPR52 shares almost identical expression profile and G-protein coupling with the striatal enriched GPR6 [237, 251]. Lately, it has been reported that N-arachidonoyl dopamine, N-docosahexaenoyl dopamine, N-oleoyl dopamine, and N-palmitoyl dopamine display inverse GPR6 agonist properties [252]. These are endogenously occurring compounds which may be enriched in striatum due to their close relationship to DA [252]. Even though there are no studies about GPR6 effects on anxiety or depression-like behaviors, this GPCR is another possible MDD drug target.

Since the discovery of LHb-RMTg aversion system, there have been attempts to selectively target the LHb to treat MDD [253]. In an effort to identify genetic tools to study LHb, GPR151 was discovered to have highly enriched expression within LHb [254–258]. Interestingly, the receptor shows extreme specificity to LHb-MHb complex as it is barely detected in other brain areas or tissue types [254–257]. Its endogenous ligand still remains unknown, however its amino-acid sequence is related to galanin receptors [259]. Additionally, it has been reported that GPR151 may exhibit some acid sensing properties [260]. As the receptor is Gα_i_ coupled and is specifically located in axons and presynaptic boutons, possible agonists may suppress habenular glutamate release [254, 257]. This is interesting in light of a recent study which has shown that intrahabenular administration of ketamine abolished burst firing of LHb neurons and induced an antidepressant-like effect mimicking the effects of the drug’s systemic administration [86]. There is an anxiety-like behavioral phenotype in mice lacking the GPR151 expressing neurons [258]. However, its role in depression-like behaviors remains to be elucidated. Consequently, future development of GPR151 agonists would allow us to pharmacologically mimic LHb DBS and offer a fast and efficient drug induced alleviation of depressive symptoms.

There are some orphan GPCRs which are highly expressed in the PFC and that have been associated with anxiety- and depression-like traits, such as GPR158, GPR56, GPR3, and GPR26. GPR158 gained attention through its remarkable upregulation in PFC in a stress-induced depression animal model but also in human patients diagnosed with MDD [261]. In spite of its presence in PFC, striatum and hippocampus, the aforementioned effect was observed solely in PFC glutamatergic cells of layer 2/3 [261]. In accordance, GPR158 is the most abundantly expressed GPCR in the PFC, making it a potential key regulator of PFC activity [261]. The receptor’s G-protein interaction properties have not been well clarified, however, there is evidence that it may be associated with RGS7 and Gα_i_ signaling [261–263]. GPR158’s ability to suppress cAMP synthesis may decrease the activity of superficial cortical neurons and thereby induce depressive behavior [261, 263]. It is indeed possible to induce a depression-like phenotype in mice via viral-induced overexpression of GPR158 in the PFC [261]. Another GPCR with altered PFC expression levels following unpredictable chronic stress is GPR56 [264]. However, unlike GPR158, deletion of GPR56 is considered to aggravate depressive-like symptoms [264].

Two brain-specific Gα_s_ coupled orphan receptors that have recently been associated with anxiety- and depression-like behaviors, are GPR3 and GPR26 [265–267]. Both GPR3 and GPR26 KO mice were shown to spend less time in the open arms of the elevated plus maze test and displayed a higher immobility time in the forced swim test [265, 267]. Regarding their brain distribution, both receptors have been localized to PFC, striatum, hippocampus and habenular complex [265, 268–271]. Remarkably, GPR26 shows notable enrichment in DA cells of VTA. GPR3 is listed in the orphan family, but presents high sequence homology with lipid binding GPCRs such as lysophospholipid and cannabinoid receptors [272, 273]. In accordance, there is some evidence that sphingosine 1 phosphate is its endogenous ligand [274].

## Chemogenetic approaches to counteract depression

Optogenetics pioneered a new era in studying functional neuroanatomical circuitries [275]. Optogenetics has been used to describe the role of different brain areas in controlling mood and depressive-like behavior. Another technique that includes the manipulation of neuronal activity with genetic tools, is chemogenetics [276–279]. The major difference between opto- and chemogenetics is that the first is using light sensitive ion channels to manipulate neuronal activity while the latter is exploiting engineered GPCRs which are stimulated selectively by specific exogenous ligands [280]. From a temporal perspective, ion channels cause rapid changes in membrane potential (<1 ms) while GPCRs initiate slower responses with longer duration (seconds–minutes) [281]. Consequently, optogenetics mimics fast neurotransmission whereas chemogenetics resembles slow neurotransmission. GPCRs used for chemogenetics are known as Designer Receptors Exclusively Activated by Designer Drugs (DREADDs). The first DREADDs were synthetic variants of muscarinic acetylcholine receptors coupled to Gα_i/o_, Gα_q/11_, or Gα_s_ proteins according to the desired functional neuronal impact. The most commonly used ligand for DREADDs is clozapine-N-oxide, which is usually administered systemically [276, 282]. Chemogenetic tools have been used to study the impact of different neuronal circuitries in regulating anxiety- and depressive-like phenotypes [276, 282]. Specifically, chemogenetic tools have been used to alter behavior in anxiety- and depression-related tests by manipulating the activity of VTA, NAc, mPFC, HF, LHb, and DRN. Chemogenetic activation of VTA abolished the anxiety and the increased immobility in FST in a genetic model of depression [283]. Suppression of NAc dMSN activity through inhibitory DREADDs causes susceptibility to social defeat in mice [49]. Chemogenetic blockade of mPFC GABAergic interneuron activity augmented learned-helplessness behavior [284]. Chemogenetic targeting of ventral HF to mPFC projecting system reduces immobility in the FST [285]. Chemogenetic inhibition of LHb complex reduced immobility time in FST [286]. At the same time, suppressing LHb neuronal activity through DREADDs rescues the stress-induced social avoidance in SSRI-resistant 5-HT deficient mice [287]. Chemogenetic activation of 5-HT cells in the DRN produces a dichotomous effect upon anxiety and depression. DREADD-induced excitation of DRN boosts anxiety but decreases depression-like behavior in mice [288].

In conclusion, chemogenetic approach in MDD animal models shows promising results for alleviating depressive-like mood. It was recently reported a successful effort to apply chemogenetics upon nonhuman primates, pushing the technology one step closer to human applications [289]. Since, gene therapy has been already used in clinical trials for PD and other disorders [290], chemogenetic strategies may be potential future treatments that will substitute DBS to specifically activate cell populations in critical brain circuitries.

## Conclusion

Over the past years several promising attempts have been made to treat MDD with receptor-based approaches. Treatment with the NMDA receptor antagonist, ketamine, is a breakthrough in the field. Rapid scientific advances in brain neuropharmacology allow the development of additional innovative approaches to treat MDD. Particularly, structural and molecular biology advances of GPCRs illuminate the possibility of pathway specific multitarget pharmacological strategies that may boost efficacy of existing drug treatments. Simultaneously, the ever-expanding knowledge of orphan GPCR function provides an opportunity for the development of first-in-class antidepressant drugs characterized by higher regional specificity and efficacy.
